# Automated closed-loop control of diabetes: the artificial pancreas

**DOI:** 10.1186/s42234-018-0015-6

**Published:** 2018-11-07

**Authors:** Boris Kovatchev

**Affiliations:** Center for Diabetes Technology, University of Virginia, P.O. Box 400888, Charlottesville, VA 22908 USA

**Keywords:** Diabetes, Hypoglycemia, Hyperglycemia, Continuous glucose monitoring (CGM), Insulin pumps, Continuous subcutaneous insulin infusion (CSII), Artificial pancreas (AP), Closed-loop control

## Abstract

The incidence of Diabetes Mellitus is on the rise worldwide, which exerts enormous health toll on the population and enormous pressure on the healthcare systems. Now, almost hundred years after the discovery of insulin in 1921, the optimization problem of diabetes is well formulated as maintenance of strict glycemic control without increasing the risk for hypoglycemia. External insulin administration is mandatory for people with type 1 diabetes; various medications, as well as basal and prandial insulin, are included in the daily treatment of type 2 diabetes. This review follows the development of the Diabetes Technology field which, since the 1970s, progressed remarkably through continuous subcutaneous insulin infusion (CSII), mathematical models and computer simulation of the human metabolic system, real-time continuous glucose monitoring (CGM), and control algorithms driving closed-loop control systems known as the “artificial pancreas” (AP). All of these developments included significant engineering advances and substantial bioelectronics progress in the sensing of blood glucose levels, insulin delivery, and control design. The key technologies that enabled contemporary AP systems are CSII and CGM, both of which became available and sufficiently portable in the beginning of this century. This powered the quest for wearable home-use AP, which is now under way with prototypes tested in outpatient studies during the past 6 years. Pivotal trials of new AP technologies are ongoing, and the first hybrid closed-loop system has been approved by the FDA for clinical use. Thus, the closed-loop AP is well on its way to become the digital-age treatment of diabetes.

## Background

On March 6, 2013, the American Diabetes Association (ADA) published research showing that the total costs of diagnosed diabetes in the U.S. have risen to $245 billion in 2012 from $174 billion in 2007, a 41% increase over 5 years (American Diabetes Association 2013). Thus, diabetes is a prime example of an enormous health care problem the only solution of which is integration of behavioral change, advanced bioengineering aiming functional replacement of the failing beta cell, and synergistic drug-device integration. Given that diabetes is common and affecting millions of people around the world, major efforts target the optimization of diabetes control and large international organizations are dedicated to the treatment and, ultimately, the cure of diabetes and its complications, including: ADA (American Diabetes Association [Internet] n.d.), the European Association for the Study of Diabetes (EASD, ( n.d.)), the International Diabetes Federation (IDF, (2014)), and the Diabetes Technology Society (Diabetes Technology n.d.). These efforts are picking up speed and new diabetes treatment technologies are being introduced daily. To put this progress in perspective, for the 1,900 years following the clinical introduction of the term *diabetes* (Aretaeus the Cappadocian, first Century AD) diet was the only treatment (albeit unsuccessful in type 1 diabetes). In the nineteenth century, the nature of diabetes was generally understood, and with the discovery of insulin in 1921 by Frederick Banting at the University of Toronto type 1 diabetes was no longer a death sentence. For this breakthrough, Banting and John Macleod were awarded the Nobel Prize in Physiology or Medicine in 1923. To recognize the contributions of their colleagues, Banting shared his prize with Charles Best and Macleod shared his with J.B. Collip.

Several subtypes of diabetes are identified, most prevalent of which are referred to as type 2 diabetes (over 90% of the people with diabetes) and type 1 diabetes – an auto-immune disorder in which the immune system targets its own β–cells in the islets of Langerhans of the pancreas – the site of insulin secretion and synthesis. Type 1 diabetes is characterized by absolute deficiency of insulin secretion, which necessitates daily (or continuous) external insulin injections to maintain carbohydrate metabolism and sustain life. Most often, type 1 diabetes occurs in childhood and adolescence (although it can occur at any age) and, until recently, was also known as “Insulin-Dependent Diabetes Mellitus (IDDM)” or “Juvenile Diabetes.” Type 2 diabetes results from a combination of impaired insulin action and insufficient β–cell function. In health, the β–cell secretes insulin in response to increase in blood glucose (BG) levels (e.g. after meals) or to elevated ambient BG. When insulin secretion is inadequate and cannot overcome the insulin resistance occurring as a result from obesity or other factors, hyperglycemia (elevated blood sugar levels) would occur. The progression of type 2 diabetes is typically gradual, beginning with pre-diabetes, i.e. impaired fasting glucose (IFG) and impaired glucose tolerance (IGT). People with type 2 diabetes are also more likely to have comorbidities, including as cardiovascular risks such as dyslipidemia and hypertension. Overall, both type 1 and type 2 diabetes require daily treatment to match insulin availability to carbohydrate intake. In type 1 diabetes this is exclusively achieved by exogenous insulin injection; in type 2 diabetes, a variety of medications are available to lower insulin resistance or amplify any residual insulin secretion; basal and prandial insulin injections are increasingly used as well.

Understanding and quantifying the dynamics of the human glucose-insulin control network is critical for the technological treatment of diabetes. BG levels are raised by food containing carbohydrates, and glucose is also produced by the body (mainly by the liver), after which it is distributed and utilized through both insulin-independent (e.g. central nervous system and red blood cells) and insulin-dependent (muscle and adipose tissues) pathways. Insulin secreted by the pancreatic β–cell is the primary regulator of glucose homeostasis. If a BG perturbation occurs (e.g. after a meal containing carbohydrates), beta-cells secrete insulin in a direct response to the increase in BG concentration, or as an indirect response to hormonal release from the gut (GIP and GLP-1, known as the “incretin effect” (Nauck et al. 1986a)). In turn, insulin signaling promotes glucose utilization and inhibits glucose production to bring rapidly and effectively plasma glucose to its pre-perturbation level (Cobelli et al. 2009). In pathophysiology, this feedback control is degraded. In type 2 diabetes, the glucose control network is largely preserved, but insulin secretion is deficient relative to hepatic and peripheral insulin resistance. In particular, the incretin response is deficient (Nauck et al. 1986b), and this finding triggered the introduction of new classes of medications known as GLP-1 receptor agonists (incretin mimetics), and DPP-4 inhibitors (incretin enhancers) (Drucker and Nauck 2006). In type 1 diabetes, insulin secretion is virtually absent, while glucagon secretion from the α–cell is still preserved, which removes the insulin-dependent pathways lowering BG levels and therefore BG can only go up, leading to hyperglycemia. Thus, insulin replacement is mandatory. In both type 1 and type 2 diabetes, a battery of counterregulatory hormones are also at work, including glucagon, epinephrine, cortisol and growth hormone, which defend the body against life-threatening low blood sugar events (i.e. severe hypoglycemia).

## The optimization problem of diabetes

**Fig. 1:**
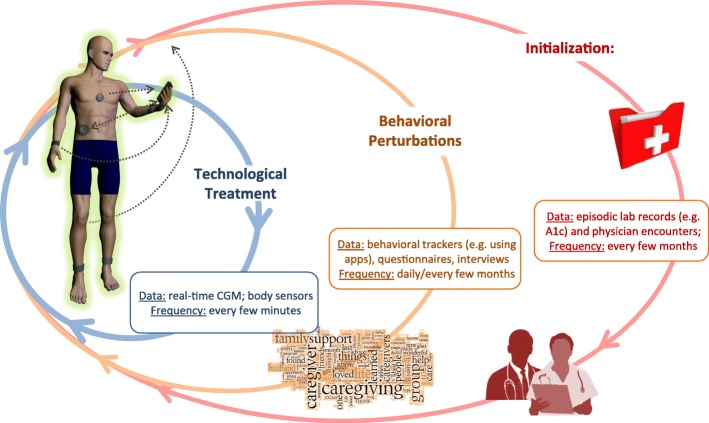
The Treatment Ecosystem of diabetes – a combination of superimposed interacting processes developing at different time scales

Classic large-scale studies have shown that intensive treatment to maintain optimal average glycemia (as measured by Hemoglobin A1c) markedly reduces the chronic complications in both type 1 (Reichard and Phil 1994; The Diabetes Control and Complications Trial Research Group 1993) and type 2 diabetes (UK Prospective Diabetes Study Group (UKPDS) 1998). External insulin replacement through multiple daily injections (MDI) or continuous subcutaneous insulin delivery (CSII) using insulin pumps, is mandatory in type 1 diabetes and is increasingly used in type 2 diabetes. However, MDI and CSII are not nearly as efficient as the endogenous insulin secretion; as a result, acute events do occur, exposing patients to severe hypoglycemia or diabetic ketoacidosis. Imperfect intensive insulin treatment may also reduce the warning symptoms and hormonal defenses against hypoglycemia leading to defective counterregulation and hypoglycemia unawareness (White et al. 1983; Cryer and Gerich 1985; Amiel et al. 1987; Amiel et al. 1988); thus, intensive treatment may trigger severe hypoglycemia (Gold et al. 1993; Henderson et al. 2003; Lincoln et al. 1996; The Diabetes Control and Complications Trial Research Group 1997). As a result, hypoglycemia has been identified as the primary barrier to optimal glycemic control (Cryer 1994; Cryer et al. 2003; Cryer 2014) and, years ago, the treatment of diabetes was clearly formulated as a “trade-off between glycemic control and iatrogenic hypoglycemia” (Cryer 2014). This means that lowering hemoglobin A1c – the primary marker of average glycemic control – must be accompanied by concurrent mitigation of the risk for hypoglycemia. Consequently, people with diabetes face a life-long optimization problem: to maintain strict glycemic control and reduce hyperglycemia, without increasing their risk for hypoglycemia. BG level is both the measurable result of this optimization and the principal feedback signal to the patient for his/her control of diabetes. This understanding of the diabetes optimization objectives led to quantitative description of the glucose-insulin control network, modeling, simulation and, ultimately, to bioengineering control of diabetes (Cobelli et al. 2009). It should be noted, however, that undesirable glycemic variation is triggered at multiple biosystem levels and is driven by self-treatment behavior. Translated to the context of contemporary diabetes technologies, such as Continuous Glucose Monitoring (CGM), Advisory Systems, or Artificial Pancreas (AP), this concept helps define a treatment ecosystem that includes several interacting processes developing at different time scales (Fig. 1). As presented in Fig. 1, with technology advancement, the focus of daily maintenance of diabetes shifts from episodic physician encounters (slowest cycle) and lab assessments (e.g. A1c measured every few months) to daily fine tuning assisted by advisory systems or automated by the AP. Each person’s behavior introduces routine (e.g. meals exercise) or abrupt (e.g. illness) perturbations. While the data at the slowest and fastest cycles are well defined (e.g. A1c and CGM), the tracking of behavioral perturbations is less common, even with contemporary applications that attempt to track human behavior. At this intermediate behavioral cycle, analytics are virtually absent; thus, the field is open for innovation and development.

The initialization of the ecosystem for a person begins with the diagnosis of diabetes, followed by choice of treatment (e.g. medication, technology) that is based on the person’s physiology as reflected by the medical record and corresponds to education, skills, or social choices. This is done infrequently. A person’s self-treatment routine (e.g. meals, exercise) is a dynamical process introducing daily behavioral perturbations to the metabolic system. These perturbations challenge the technological treatment of diabetes, which includes real-time warning, alarms, advisory systems, or automated insulin delivery. Thus, the key to optimal diabetes treatment is a multi-layer holistic approach, which must use advanced bioelectronics and technology to account for individual factors of human physiology and behavior. The optimization of diabetes must rely on an entire bio-behavioral treatment ecosystem of signals, models, and control methods that act at several levels of biosystem organization – from metabolic to human behavior and social interaction.

## A century of diabetes technology

**Fig. 2:**
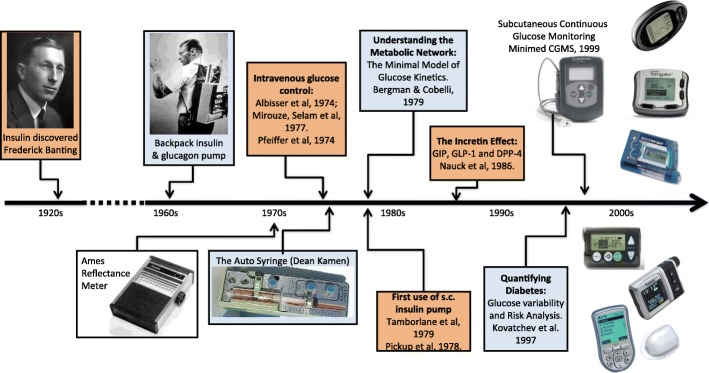
Timeline of diabetes technology development following the discovery of insulin in 1921

In the early 60’s, an intravenous (i.v.) insulin pump delivering insulin and glucagon to counteract hypoglycemia was reported by Kadish (1964). In 1969, the first portable blood glucose meter – the Ames Reflectance meter - was manufactured. The first commercial subcutaneous insulin pump – the Auto Syringe - was introduced by Kamen in the 1970s, and by the end of the 70’s the first trials of continuous subcutaneous insulin infusion (CSII) were reported by Pickup et al. in the U.K. (Pickup et al. 1978) and Tamborlane et al. in the U.S. (Tamborlane et al. 1979). These devices demonstrated the feasibility of ambulatory glucose measurement and external, including subcutaneous, insulin delivery. The next step was to automate the process of insulin replacement in type 1 diabetes – from BG monitoring to insulin delivery controlled by a mathematical algorithm. This approach became known as closed-loop control of diabetes, or the “artificial pancreas (AP).” The AP idea can be traced back to the 1970’s when the possibility for external blood glucose regulation was established by studies using i.v. glucose measurement and i.v. infusion of glucose and insulin (Albisser et al. (1974), Pfeiffer et al. (1974), Mirouze et al. (1977), Kraegen et al. (1977), and Shichiri et al. (1978)). In 1977, one of these designs (Pfeiffer et al. 1974) resulted in the first commercial device - the Biostator (Clemens et al. 1977) - a large (refrigerator-sized) device that has been used extensively for glucose-control research (Marliss et al. 1977; Santiago et al. 1979; Fischer et al. 1978). A review of the methods for i.v. glucose control developed in the 70’s can be found in (Parker et al. 2001). In 1979, another key element – the Minimal Model of Glucose Kinetics – was introduced by Bergman and Cobelli (Bergman et al. 1979). This, and subsequent mathematical models, serve as the “brain” behind the majority of control algorithms used in contemporary artificial pancreas systems. Detailed description of the major early algorithm designs can be found in (Broekhuyse et al. 1981; Clemens 1979; Cobelli and Ruggeri 1983; Salzsieder et al. 1985). More work followed, spanning a range of control techniques powered by physiologic modeling and computer simulation (Brunetti et al. 1993; Fischer et al. 1987; Sorensen 1985; Parker et al. 1999). Between 1980 and 2000 the insulin pumps became smaller and portable, while the models of the glucose-insulin system became larger and more elaborate, allowing first computer simulation and then automated model-predictive glucose control of diabetes. In 1997–1998, the first elements of the risk analysis of BG data were introduced (Kovatchev et al. 1997; Kovatchev et al. 1998), which later became the base for closed-loop safety systems embedded in the AP design. Figure 2, which was first published in (Kovatchev 2018a), presents the timeline of these developments:

## Continuous glucose monitoring

The final critical technological leap enabling minimally-invasive closed-loop was made at the turn of the twenty-first century with the introduction of CGM devices by Medtronic, Abbott, Dexcom, Cygnus, and others (Mastrototaro 2000; Bode 2000; Feldman et al. 2003). It is important to know, however, that CGM devices measure glucose concentration in a different compartment – the interstitium – which introduces an additional (presumably diffusion) process between blood and interstitial glucose (IG) (Rebrin et al. 1999; Rebrin and Steil 2000; Steil et al. 2005). To account for the gradient between BG and IG, CGM devices are typically calibrated with capillary BG readings. Successful calibration would adjust the amplitude of IG fluctuations with respect to BG, but would only partially mitigate the time lag due to BG-to-IG glucose transport. Because such a time lag could greatly influence the accuracy of CGM, a number of studies were dedicated to its investigation (Boyne et al. 2003; Kulcu et al. 2003; Stout et al. 2004; Wentholt et al. 2004). It was hypothesized that if a glucose fall is due to peripheral glucose consumption, the physiologic time lag would be negative, i.e. fall in IG would precede fall in BG (Rebrin et al. 1999; Wientjes and Schoonen 2001). But, in most studies, IG lagged behind BG by 4–10 min, regardless of the direction of BG change (Steil et al. 2005; Boyne et al. 2003). In an attempt to reconcile these results, the formulation of the push-pull phenomenon offered arguments for a more complex BG-IG relationship than a simple constant or directional time lag (Wentholt et al. 2004; Aussedat et al. 2000; Basu et al. 2013; Basu et al. 2015). In addition, errors from calibration, loss of sensitivity, and random noise confound CGM data (Kovatchev and Clarke 2008). Nevertheless, the accuracy of CGM is increasing (Clarke and Kovatchev 2007; The Diabetes Research in Children Network (DirecNet) Study Group 2008; Kovatchev et al. 2008; Garg et al. 2009; Christiansen et al. 2013) and has reached a point where CGM could be used as a replacement to traditional BG measurement without calibration with capillary blood several times a day (Kovatchev et al. 2015). For example, a few years ago the Dexcom G4 Platinum and G5 CGMs used algorithmic signal processing to improve its accuracy and obtain replacement clearance from the Food and Drug Administration (FDA) (Facchinetti et al. 2013; Peyser et al. 2015; Kovatchev 2015). Most recently, the new version of these devices – Dexcom G6 – was approved by the FDA for use without fingerstick calibration.

## Closed-loop control

In addition to presenting frequent data (e.g. every 5–10 min), real-time CGM devices typically display trends, BG rate of change, and alerts for upcoming hypo- or hyperglycemia (Heise et al. 2003; Bode et al. 2004; McGarraugh and Bergenstal 2009). As a result, a number of studies have documented the benefits of CGM technology (Deiss et al. 2006; Garg et al. 2006; Kovatchev and Clarke 2007; The Juvenile Diabetes Research Foundation Continuous Glucose Monitoring Study Group 2008) and envisioned its use in closed-loop control systems (Klonoff 2005; Hovorka 2006; Klonoff 2007). The first step in this direction was the introduction of a system attempting to prevent hypoglycemia via automated shutoff of the insulin pump when CGM readings crossed a predetermined low glucose threshold (Buckingham et al. 2009) – a feature that became known as Low Glucose Suspend (LGS) - or Predictive Low Glucose Suspend (PLGS) in implementations which used forecast of glucose values. First steps towards fully automateing the glucose control in diabetes using CGM and CSII linked via a closed-loop control algorithm, were taken by the early work of Hovorka et al. (2004a) and Steil et al. (2006). The launch of the JDRF AP Consortium in 2006, which sponsored several centers in the U.S. and Europe to carry closed-loop control research, institutionalized this trend. In 2009 *JAMA* wrote: “*Artificial pancreas may soon be a reality*” (Friedrich 2009). In May 2012, a Diabetes Outlook was published in *Nature* (Dolgin 2012) which highlighted the AP, and 18 months later *Science* featured the same topic (Clery 2014). In 2008 the National Institutes of Health (NIH) launched an AP initiative and in 2010 the European AP@Home Consortium was established. A roadmap towards a viable AP was accepted, which included several sequential steps, beginning with automated mitigation of hypoglycemia and progressing through control-to-range and control-to-target towards fully automated, possibly multi-hormonal AP (Kowalski 2009). By 2010 the AP became a global research topic engaging physicians and engineers in an unprecedented collaboration. In 2014, *JAMA* revisited the AP and suggested: “*Fully automated artificial pancreas finally within reach*” (Hampton 2014). Key milestones of this development are described in our reviews (Cobelli et al. 2011; Kovatchev 2011; Renard et al. 2013a; Renard et al. 2013b).

### The control algorithm

A key element of the AP system is the control algorithm, which monitors BG fluctuations and the actions of the insulin pump, and computes insulin delivery rate every few minutes (Bellazzi et al. 2001). The first studies of Hovorka et al. (2004a; 2004b) and Steil et al. (2006) outlined the two major types of closed-loop control algorithms now in use – model-predictive control (MPC, (Hovorka et al. 2004b)) and proportional-integral-derivative (PID, (Steil et al. 2006)). By 2007, the blueprints of the contemporary controllers were in place, including run-to-run control (Zisser et al. 2005; Owens et al. 2006; Palerm et al. 2007) and linear MPC (Magni et al. 2007). MPC became the approach of choice targeted by recent research, for two main reasons: (i) PID is purely reactive, responding to changes in glucose level, while a properly tuned MPC allows for prediction of glucose dynamics and, as a result, for mitigation of the time delays inherent with subcutaneous glucose monitoring and subcutaneous insulin infusion; (ii) MPC allows for relatively straightforward personalizing of the control using patient-specific model parameters. In addition, MPC could have “learning” capabilities - it has been shown that a class of algorithms (known as run-to-run control) can “learn” specifics of patients’ daily routine and then optimize the response using this information. A combination of MPC and PID was used to drive a dual-hormone AP system that added glucagon to combat hypoglycemia (El-Khatib et al. 2010a). A new step towards adaptive AP was recently taken by a12-week multi-center trial of 24/7 personalized closed-loop control (Dassau et al. 2017). In this trial, each participant’s insulin requirements (e.g. basal rate settings, carbohydrate ratio) were algorithmically adapted every week.

### Inpatient AP studies

During 2008–2012, promising results were reported by several groups (Weinzimer et al. 2008; Clarke et al. 2009; Bruttomesso et al. 2009; Hovorka et al. 2010; El-Khatib et al. 2010b; Renard et al. 2010; Hovorka et al. 2011; Zisser et al. 2011; Russell et al. 2012; Breton et al. 2012; Luijf et al. 2013; Zisser et al. 2014; Sherr et al. 2013). Most of these studies pointed out the superiority of closed-loop control over CSII therapy in terms of: (i) increased time within target range (typically 70–180 mg/dl); (ii) reduced incidence of hypoglycemia, and (iii) better overnight control. Inpatient studies linked AP to reduction of hypoglycemia following exercise (Breton et al. 2012; Zisser et al. 2014; Sherr et al. 2013), and an auxiliary heart rate signal was shown to contribute to better AP adaptation to exercise (Breton et al. 2014). An algorithm mimicking β-cell physiology was embedded in a low-power microchip (Reddy et al. 2014), and an MPC control algorithm running on a smart phone was tested in the hospital (O’Grady et al. 2012). A direct comparison between single- vs. dual-hormone AP found “*little evidence of glucagon benefits in reducing the number of hypoglycemic events requiring treatment*” (Haidar et al. 2015) – an important finding that helped settle the debate whether dual-hormone AP using glucagon is justified, given the added complexity, possible risks, and expenses.

### AP system integration

LGS, which is now commercially available and is already a part of the clinical practice, is considered a precursor to AP because of the automated data transfer from CGM to the insulin pump – a system integration that was a critical step in the AP development. The ASPIRE trial showed a 38% reduction in nocturnal hypoglycemia compared to CGM alone without increasing HbA1c (Bergenstal et al. 2013); a subsequent study achieved similar results (Ly et al. 2013). PLGS algorithms were introduced (Beck et al. 2014), which brought this type of system to a higher level of computational sophistication. However, LGS and PLGS systems are still binary on-off insulin “switches;” they lack the defining property of a closed-loop algorithm – feedback estimation of the patient state using CGM and insulin delivery data to determine the degree or insulin modulation in real time.

### Outpatient (but not portable) AP

The first steps towards outpatient AP were taken by a laptop-based system installed at the bedside of children at a diabetes camp (Phillip et al. 2013), and then taken to patients’ homes (Nimri et al. 2013). Similarly, another bedside AP trial deployed small personal computers at patients’ homes, and confirmed their feasibility outside of the hospital (Hovorka et al. 2014). The progress of the outpatient AP was presented in a symposium “Advances in Artificial Pancreas Development” (*Diabetes Care*, May 2014), which contained an editorial (Cefalu and Tamborlane 2014) and 7 papers on topics ranging from physiology and engineering (Kudva et al. 2014; Schiavon et al. 2014) to reports on predictive LGS (Beck et al. 2014), overnight AP at home (Hovorka et al. 2014), feasibility of the AP in type 2 diabetes (Kumareswaran et al. 2014), and around-the-clock outpatient AP with a portable AP system (Del Favero et al. 2014). Subsequent studies firmly took the AP to outpatient setting (Russell et al. 2014; Leelarathna et al. 2014; Thabit et al. 2014).

## Wearable artificial pancreas

In the *Nature* (Dolgin 2012) and *Science* (Clery 2014) reviews cited above, a common photo appeared of a smart phone presenting a dual traffic-light display – the face of the first portable AP platform–the *Diabetes Assistant (DiAs*) developed at the University of Virginia (UVA) in 2011. DiAs was built using an Android smart phone as a computational hub and, for the first time, included two features now employed by virtually all outpatient AP systems: (i) graphical user interface designed for the patient (Keith-Hynes et al. 2013), and (ii) a Web-based remote monitoring system (Place et al. 2013). The defining characteristic of DiAs was its capability to switch between different modes of operation, depending on patient preference and signal availability. For example, if CGM signal were not available or if the patient does not wear a sensor for a certain period of time, DiAs could switch into “pump mode” running this patient’s normal basal/bolus insulin routine and adding extended GUI and remote monitoring. Similarly, if the pump were unavailable, DiAs could switch to “sensor mode” running trends, alerts, and Cloud services. Thus, by design, DiAs was a platform for AP technology deployment, used in 20 clinical trials, by over 300 patients at clinical centers in the U.S. (Virginia, California, Minnesota, and New York), as well as in France, Italy, Holland, Israel, and Argentina. Some examples: Following initial 2-day pilot-feasibility trials (Cobelli et al. 2012), in 2012–2013 two international multi-site studies were completed, which confirmed the feasibility of DiAs and its efficacy to reduce hypoglycemia in the outpatient setting (Kovatchev et al. 2013; Kovatchev et al. 2014). Three summer camp trials of remote monitoring (DeSalvo et al. 2013), overnight AP (Ly et al. 2014), and AP during the day (Chernavvsky et al. 2016) confirmed the efficacy of DiAs in children with type 1 diabetes. With several completed multi-center trials, smart-phone based closed-loop control is now considered an established and effective approach to portable AP (Brown et al. 2015a; Del Favero et al. 2015; Brown et al. 2015b; Anderson et al. 2015; Renard et al. 2015).

**Fig. 3:**
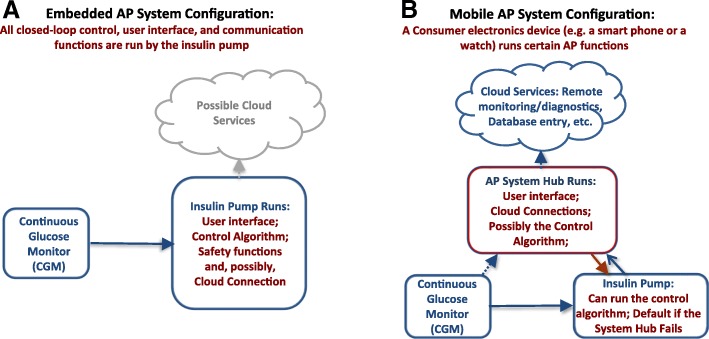
The two principal configurations of a wearable artificial pancreas: embedded, with a control algorithm running on board of an insulin pump, and mobile, using consumer electronics as a computational and communication hub. Both configurations have their advantages and disadvantages

The studies done in the past few years ascertained the two principal implementations of a wearable AP – a system *embedded* in the insulin pump and a *mobile* system based on a smart phone. These system configurations are presented in Fig. 3, which was first published in *Nature Reviews Endocrinology* in a visually different, but conceptually similar form (Kovatchev 2018b).

Each of the configurations presented in Fig. 3 has advantages and disadvantages, and both are likely to coexist as means for automated insulin delivery. Embedded systems have fewer components and fewer wireless connections and, theoretically, should be more reliable that a consumer electronics device such as a smart phone. However: (i) smart phones are inexpensive, available, computationally able to run even complicated control algorithms, and wirelessly connectable to various devices and data networks–no current insulin pump offers similar capabilities; (ii) the life cycle of a smart phone is months, as opposed to years for insulin pumps; thus, smart phones allow easier updates of user interface and device form factor; (iii) psychologists share that patients, particularly youth, miss boluses because of reluctance to use their insulin pump in public; but, no one is reluctant to use a phone and that may be a key to AP success and widespread adoption. Thus, the last remaining question is whether an AP system based on consumer electronics could receive regulatory clearance from the FDA, and this subject is intensively discussed now.

The progress of the artificial pancreas was presented in a symposium published by *Diabetes Care* in July 2016 (Kovatchev et al. 2016), which was exclusively dedicated to outpatient closed-loop control studies done with portable AP systems (Anderson et al. 2016; Renard et al. 2016; Tauschmann et al. 2016; Del Favero et al. 2016), including trials at patient’s homes lasting a month or more (Anderson et al. 2016; Renard et al. 2016; Tauschmann et al. 2016) and studies in young children (Del Favero et al. 2016). Recently, I summarized the progress made in 2017 – the year of transition of the AP to everyday clinical use (Kovatchev 2018b) – as follows:

1. A pivotal trial of the first commercial hybrid CLC system was completed – the Medtronic 670G which modulates automatically basal rate but does not automate insulin boluses. Following the initial summary published in *JAMA (Bergenstal* et al. 2016*)*, Garg et al. (2017) reported detailed glycemic control outcomes from the use of this system in adolescents (ages 14–21 years) and adults. This study allowed the comprehensive testing the safety of in-home use of hybrid CLC and the subsequent regulatory approval of the system by the U.S. Food and Drug Administration (FDA), thereby opening the AP field to routine clinical use;
2. A six-month pilot-feasibility study of long-term closed-loop control was reported showing improvements in glycemic control and simultaneous reduction of hypoglycemia with long-term AP use (Kovatchev et al. 2017);
3. Long-term (up to 10 months) studies were initiated using a successor of the DiAs system – inControl AP (TypeZero Technologies), which runs on android smart phone and connects wirelessly to a CGM sensor and an insulin pump;
4. The National Institutes of Health invested over $35 M in four pivotal trials of closed-loop control technologies intended to bring these systems to market – a press release can be found at https://www.nih.gov/news-events/news-releases/four-pivotal-nih-funded-artificial-pancreas-research-efforts-begin,, and
5. The first AP Ski Camp and was successfully completed indicating that the use of closed-loop control in the conditions of winter sports (5 h of skiing per day for 5 days) is safe and effective for children and adolescents with type 1 diabetes (Breton et al. 2017).

## Conclusions

The Artificial Pancreas technology is indeed within reach, and *is* the ultimate bioelectronics approach to improve glucose control in diabetes. In the process of AP development, new technologies were developed, together with sophisticated models of the human metabolic system. Continuous glucose monitoring and insulin pumps are now mainstream. The 2008 FDA acceptance of the UVA/University of Padova computer simulator as a substitute to animal trials in the design and safety pre-clinical testing of AP controllers opened the field for rapid and cost-effective in silico experiments (Kovatchev et al. 2009a). Since then, no animal experiments have been conducted for the purpose of designing AP algorithms. It can be envisioned that virtual environments will increasingly allow thorough testing of myriad control cycles in extreme physiological situations, or during low-probability system failures, that cannot be reproduced in real life (Viceconti et al. 2017). Modular architecture allows AP systems to be assembled from independent (but compatible) modules, each performing a specific function, e.g. prevention of hypoglycemia, post-meal insulin corrections, fine tuning of basal rate, or administration of ancillary compounds such as amylin or glucagon (Kovatchev et al. 2009b). Modularity is essential for the “graceful degradation” of the system in the event of component failure and permits critical functions to be redundant, residing in more than one module, and redirected to avoid system meltdown (Patek et al. 2012).

Two main AP system configurations have emerged: embedded, running a control algorithm in the insulin pump and mobile, using a smart phone as a data processing and communication device. Traditionally, it was assumed that the AP control system would reside in a patient’s insulin pump, which is the case with most commercial developments. An alternative solution was suggested by DiAs, which was based on appropriately configured consumer electronic devices (smart phone). This approach offered certain advantages to the initial research phase of AP development, and may be potentially viable for commercial systems as well.

In conclusion, to be ultimately established and accepted as a viable treatment of diabetes, AP systems need to prove their safety and efficacy in large-scale clinical trials with outcomes tied to the key components of glycemic control in diabetes – hemoglobin A1c and risk for hypoglycemia. These validations are ongoing now; thus, the optimism is high for the closed-loop artificial pancreas becoming soon the digital-age bioelectronics approach to the treatment of diabetes.

## Abbreviations

ADA: American Diabetes Association
AP: Artificial Pancreas
BG: Blood Glucose
CGM: Continuous Glucose Monitoring
CSII: Continuous Subcutaneous Insulin Infusion
DiAs: Diabetes Assistant
EASD: European Association for the Study of Diabetes
FDA: Food and Drug Administration
GIP: Glucose-dependent Insulinotropic Peptide
GLP-1: Glucagon-like Peptide-1
IDDM: Insulin-Dependent Diabetes Mellitus
IDF: International Diabetes Federation
IFG: Impaired Fasting Glucose
IG: Interstitial Glucose
IGT: Impaired Glucose Tolerance
LGS: Low Glucose Suspend
MDI: Multiple Daily Injections
MPC: Model-Predictive Control
NIH: National Institutes of Health
PID: Proportional-Integral-Derivative
PLGS: Predictive Low Glucose Suspend
UVA: University of Virginia
